# Supplementary material for: An EGFR/HER2-targeted conjugate sensitizes gemcitabine-sensitive and resistant pancreatic cancer through different SMAD4-mediated mechanisms
Source: Nat Commun. 2022 Sep 20;13:5506. doi: 10.1038/s41467-022-33037-x (PMC9489697; doi:10.1038/s41467-022-33037-x)
Supplement: Supplementary file 1 — Supplementary Info [file 41467_2022_33037_MOESM1_ESM.pdf]

Supplementary Information for

An EGFR/HER2-targeted conjugate sensitizes gemcitabine-sensitive and resistant pancreatic cancer through different SMAD4-mediated mechanisms

Supplementary Figure 1……………………………………………………… …….. 2
Supplementary Figure 2…………………………………………………………...3
Supplementary Figure 3…………………………………………………………4
Supplementary Figure 4…………………………………………………………5
Supplementary Table 1………………………………………………………………….. 6
Supplementary Table 2………………………………………………………………….. 7
Supplementary Table 3 ………………………………………………………………… 8
Supplementary Table 4………………………………………………………………….. 9
Supplementary Table 5………………………………………………………………….. 9

a

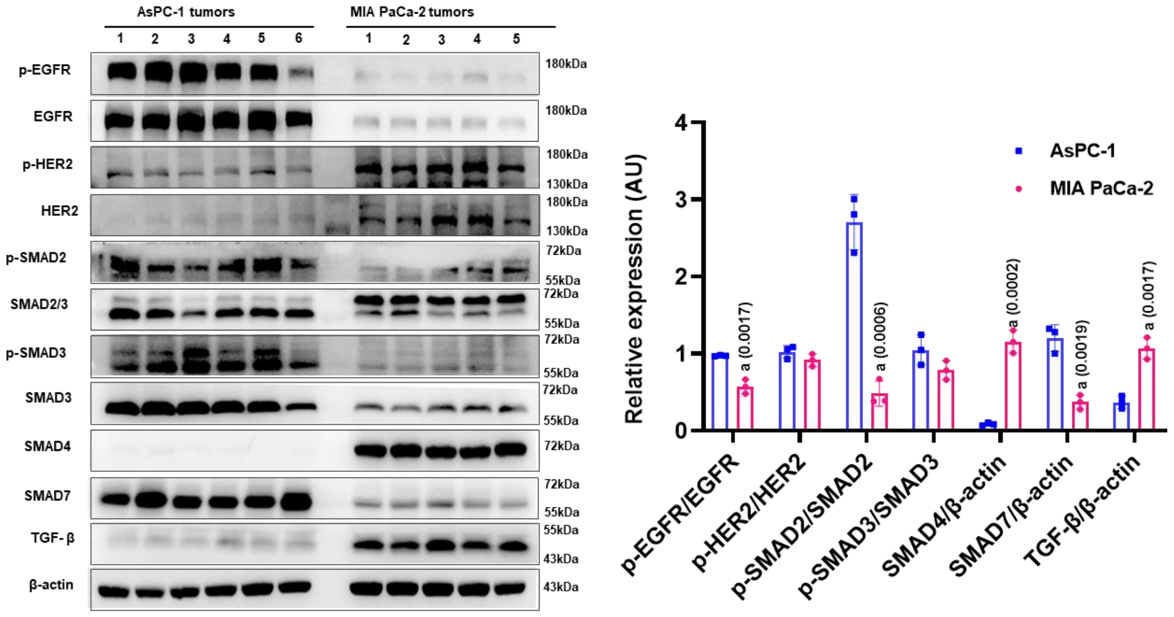

b

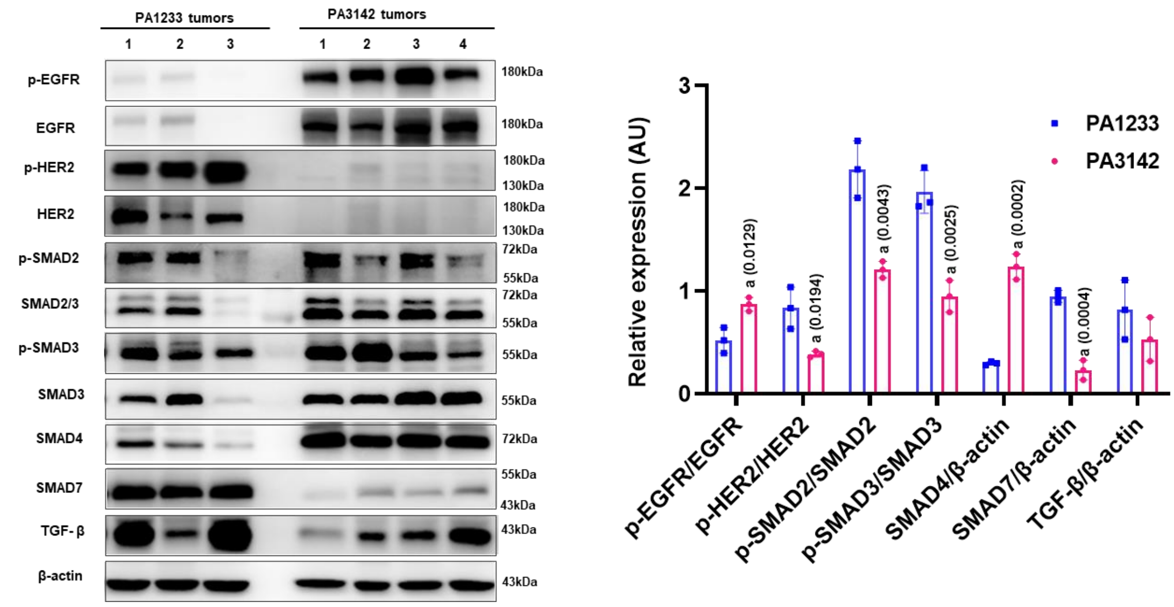

Supplementary Figure 1. Basal protein levels of tumor tissue samples were determined in Western blot assays by using either CDX (a) or PDX (b) models. The protein levels of EGFR, HER2, SMAD2, SMAD3, SMAD4, SMAD7 and TGF-β in tumor tissue without treatment were detected by Western blot analysis. Band intensities were quantified using Image J. Data are representative of biologically independent replicates as the mean ±SD (n = 6 for AsPC-1 tumors, n = 5 for MIA PaCa-2 tumors, n = 3 for PA1233 tumors and n =4 for PA3142 tumors). Note: ‘a’ indicates $p<0.05$ as compared with the AsPC-1 xenografts model or PA1233 model. Two-sided, paired-samples t-test was used in Supplementary Figure 1a, 1b. All significant p values are shown in parentheses behind letter “a”. Source data are provided as a Source Data file.

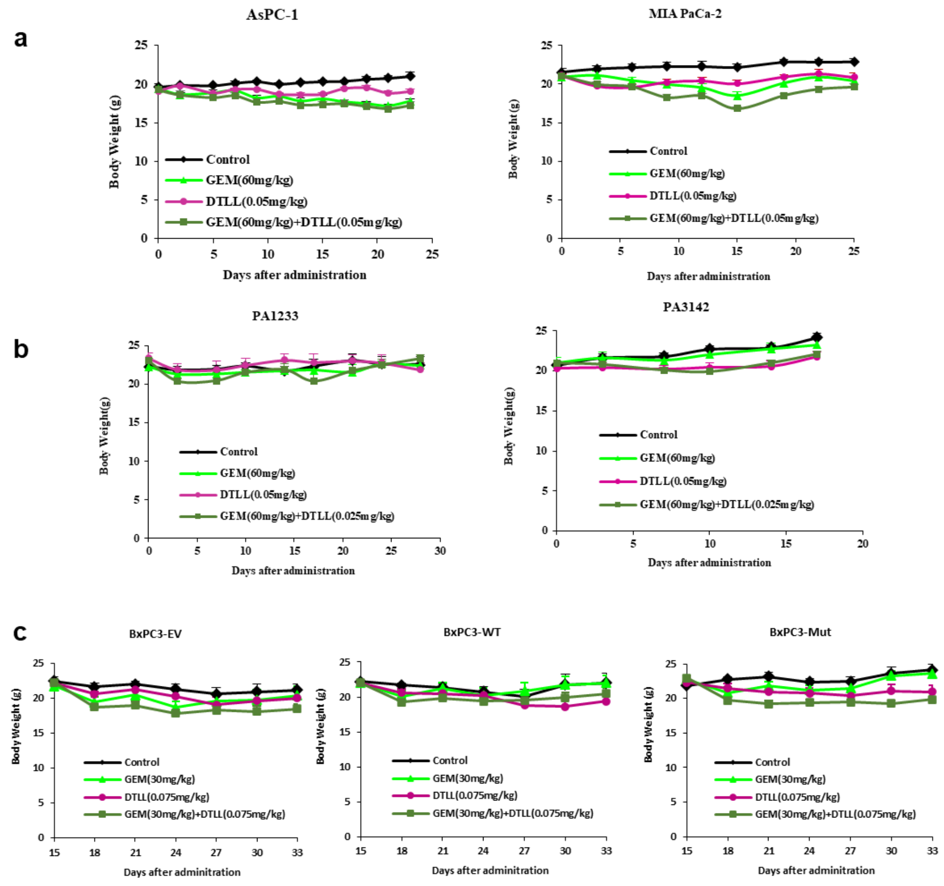

Supplementary Figure 2. Changes in mouse body weight in AsPC-1 and MIA PaCa-2 cell line-derived xenograft (CDX) models (a), patient-derived xenograft (PDX) models (b) and BxPC3-EV, BxPC3-WT and BxPC3-Mut stably transfected cell line-derived xenograft (CDX) models (c) after treatment with GEM, DTLL or both drugs. In AsPC-1 and MIA PaCa-2 xenograft models, the mice were received equal volume of physiological saline (Control group), 60 mg/kg gemcitabine intraperitoneally administered once a week (GEM group), 0.05 mg/kg DTLL at the LDM-equivalent dose intravenously administered every ten day (DTLL group) and 60 mg/kg gemcitabine combining with 0.05 mg/kg DTLL (GEM +DTLL group); In PA1233 and PA3142 models, the mice were received equal volume of physiological saline (Control group), 60 mg/kg gemcitabine every four day (GEM group), 0.05 mg/kg DTLL once a week (DTLL group) and 60 mg/kg gemcitabine combining with 0.025 mg/kg DTLL (GEM + DTLL group). In those three BxPC-3 xenografts models, the mice were received equal volume of physiological saline (Control group), 30 mg/kg gemcitabine intraperitoneally administered every four day (GEM group), 0.075 mg/kg DTLL at the LDM-equivalent dose intravenously administered once a week (DTLL group) and 30 mg/kg gemcitabine combining with 0.075 mg/kg DTLL (GEM+DTLL group). Data are representative of biologically independent replicates as the mean ±SEM (n =6 for AsPC-1 tumors, n =5 for MIA PaCa-2 tumors, n =3 for PA1233 tumors, n =4 for PA3142 tumors and n =5 for BxPC3 tumors). Source data are provided as a Source Data file.

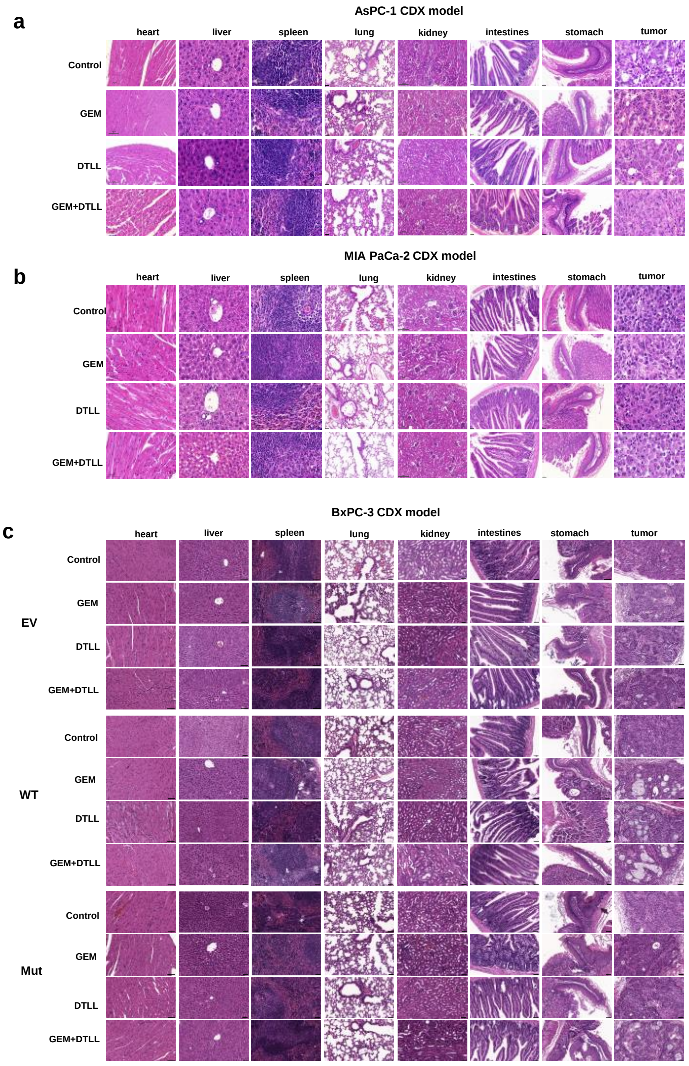

Supplementary Figure 3. Histopathological examination for tumor tissues and various organs of AsPC-1 (a), MIA paca-2(b) and BxPC3 cells with stable expression of wild-type and mutant SMAD4 (c) xenograft-bearing mice treated with GEM, DTLL and both (H & E staining, ×200)**.** The sections of tumor tissue and major organs, including the heart, liver, spleen, lung, kidney, intestines, and stomach were stained by the routine hematoxylin–eosin staining method, and then were photographed for the histopathology assay. Scale bars indicate 50 μm. In AsPC-1 and MIA PaCa-2 xenografts model, the mice were received equal volume of physiological saline (Control group), 60 mg/kg gemcitabine intraperitoneally administered once a week (GEM group), 0.05 mg/kg DTLL at the LDM-equivalent dose intravenously administered every ten day (DTLL group) and 60 mg/kg gemcitabine combining with 0.05 mg/kg DTLL (GEM+DTLL group). In those three BxPC-3 xenografts models, the mice were received equal volume of physiological saline (Control group), 30 mg/kg gemcitabine intraperitoneally administered every four day (GEM group), 0.075 mg/kg DTLL at the LDM-equivalent dose intravenously administered once a week (DTLL group) and 30 mg/kg gemcitabine combining with 0.075 mg/kg DTLL (GEM+DTLL group). Images are representative of three biologically independent replicates with a scale bar representing 50μm. Source data are provided as a Source Data file.

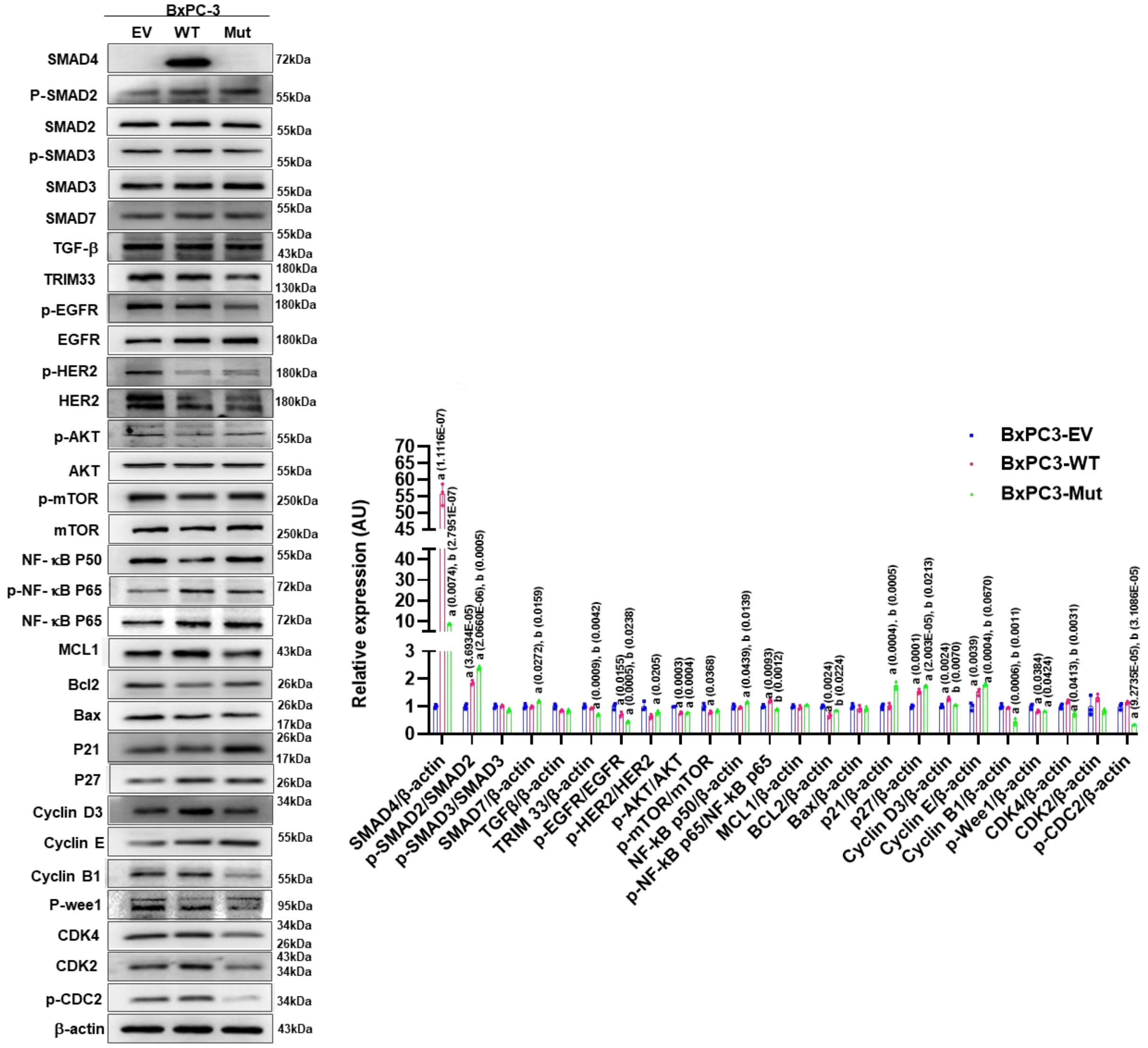

Supplementary Figure 4. Difference in protein expression levels in BxPC3-EV, BxPC3-WT and BxPC3-Mut stably transfected cells. The protein expression of SMAD4, SMAD2, SMAD3, SMAD7, TGF-β, TRIM33, EGFR, HER2, AKT, mTOR, NF-κB, MCL1, Bcl2, BAX, P21, P27, Cyclin D, Cyclin E, Cyclin B, p-Wee1, CDK2, CKK4 and p-CDC2 were detected by Western blot analysis. Data are shown as mean ± SD from three biologically independent samples (n =3). Note: 'a' indicates p<0.05 as compared with the BxPC3-EV group and 'b', p<0.05 compared with the BxPC3-WT group**.** One-way ANOVA with Bonferroni post hoc test was used. All significant P values are shown in parentheses behind letter "a" or "b". Source data are provided as a Source Data file.

Supplementary Table 1. Therapeutic efficacy of the combination of DTLL and GEM against pancreatic carcinoma in both CDX and PDX mouse models

Models	Treatment groups	Number	Tumor volume (mm^3) Mean±SEM	Inhibition rate (%) Mean±SEM
AsPC-1	Control	6	1079.31±80.72	-
	GEM(60mg/kg)	6	798.12±55.38	26.05±5.13[a]
	DTLL (0.05mg/kg)	6	649.76±40.80	39.80±3.78[a]
	GEM(60mg/kg) + DTLL (0.05mg/kg)	6	356.63±37.92	66.96±3.51[a,b, c]
MIA PaCa-2	Control	5	1538.97±172.35	-
	GEM(60mg/kg)	5	630.32±63.60	59.04±4.13[a]
	DTLL (0.05mg/kg)	5	313.57±63.86	79.62±3.79[a,b]
	GEM(60mg/kg) + DTLL (0.05mg/kg)	5	182.43±34.83	88.15±2.09[a,b]
PA1233	Control	3	719.47±108.21	-
	GEM(60mg/kg)	3	635.64±87.28	11.65±12.13
	DTLL (0.05mg/kg)	3	489.03±131.00	31.54±18.21
	GEM(60mg/kg) + DTLL (0.025mg/kg)	3	199.68±79.24	72.25±11.01[a,b]
PA3142	Control	4	2653.11±54.20	-
	GEM(60mg/kg)	4	1210.63±224.30	54.37±8.45[a]
	DTLL (0.05mg/kg)	4	1313.27±184.69	50.50±6.96[a]
	GEM(60mg/kg) + DTLL (0.025mg/kg)	4	605.65±129.37	77.17±16.95[a,c]

In AsPC-1 and MIA PaCa-2 xenograft models, the mice were received equal volume of physiological saline (Control group), 60 mg/kg gemcitabine intraperitoneally administered once a week (GEM group), 0.05 mg/kg DTLL at the LDM-equivalent dose intravenously administered every ten day (DTLL group) and 60 mg/kg gemcitabine combining with 0.05 mg/kg DTLL (GEM +DTLL group). In PA1233 and PA3142 models, the mice were received equal volume of physiological saline (Control group), 60 mg/kg gemcitabine every four day (GEM group), 0.05 mg/kg DTLL once a week (DTLL group) and 60 mg/kg gemcitabine combining with 0.025 mg/kg DTLL (GEM + DTLL group). Data are representative of biologically independent replicates as the mean ± SEM. Note: 'a' indicates $p<0.05$ as compared with the control, 'b', $p<0.05$ compared with the GEM group and 'c', $p<0.05$ compared with the DTLL group**.** In either AsPC-1 or MIA PaCa-2 CDX models, $p<0.001$ for 'GEM+DTLL' versus Control, 'GEM+DTLL' versus GEM, DTLL versus Control and GEM versus Control. In AsPC-1 CDX model, $p<0.001$ for 'GEM+DTLL' versus DTLL. In PA3142 model, $p<0.001$ for 'GEM+DTLL' versus Control. One-way ANOVA with Bonferroni test was used. Source data are provided as a Source Data file.

Supplementary Table 2. Therapeutic efficacy of the combination of DTLL and GEM against pancreatic carcinoma in CDX mouse models derived from BxPC-3 cells with SMAD4 stable overexpression vectors

Models	Treatment groups	Number	Tumor volume (mm^3) Mean±SEM	Inhibition rate (%) Mean±SEM
BxPC3-EV	Control	5	731.31±65.53	-
	GEM (30mg/kg)	5	522.44±24.90	28.56±3.41[a]
	DTLL (0.075mg/kg)	5	467.38±39.07	36.09±5.34[a]
	GEM(30mg/kg)+DTLL (0.075mg/kg)	5	282.88±18.95	61.32±2.59[a,b,c]
BxPC3-WT	Control	5	1118.80±67.14	-
	GEM(30mg/kg)	5	387.99±22.89	65.32±2.05[a]
	DTLL (0.075mg/kg)	5	456.62±50.34	59.19±4.50[a]
	GEM(30mg/kg) + DTLL (0.075mg/kg)	5	200.83±17.67	82.05±1.58[a,b,c]
BxPC3-Mut	Control	5	956.12±63.07	-
	GEM(30mg/kg)	5	884.98±51.70	7.44±5.41
	DTLL (0.075mg/kg)	5	500.99±31.39	47.60±3.28[a,b]
	GEM(30mg/kg) + DTLL (0.075mg/kg)	5	204.57±24.22	78.60±2.53[a,b,c]

In those three BxPC-3 xenografts models, the mice were received equal volume of physiological saline (Control group), 30 mg/kg gemcitabine intraperitoneally administered every four day (GEM group), 0.075 mg/kg DTLL at the LDM-equivalent dose intravenously administered once a week (DTLL group) and 30 mg/kg gemcitabine combining with 0.075 mg/kg DTLL (GEM+DTLL group). Data are representative of biologically independent replicates as the mean ± SEM. Note: 'a' indicates $p<0.05$ as compared with the control, 'b', $p<0.05$ compared with the GEM group and 'c', $p<0.05$ compared with the DTLL group**.** In BxPC3-EV, BxPC3-WT and BxPC3-Mut models, $p<0.001$ for 'GEM+DTLL' versus Control, 'GEM+DTLL' versus DTLL and DTLL versus Control. In BxPC3-EV model, $p<0.001$ for 'GEM+DTLL' versus GEM and GEM versus Control. In BxPC3-WT model, $p<0.001$ for GEM versus Control. In BxPC3-Mut model, $p<0.001$ for 'GEM+DTLL' versus GEM. One-way ANOVA with Bonferroni test was use. Source data are provided as a Source Data file.

Supplementary Table 3. Twelve detectable protein levels of gemcitabine-relevant transporters or pharmacokinetic enzymes in BxPC3-Mut and BxPC3-WT cells with treatments of control, gemcitabine, DTLL and both drugs with treatments of control, gemcitabine, DTLL and both drugs in proteomics analysis.

PG. Genes	PG. Protein Accessions ID	BxPC3-Mut_Control vs BxPC3-WT_Control		BxPC3-Mut cells						BxPC3-WT cells					
				Gem vs Control		DTLL vs Control		DTLL+Gem vs Control		Gem vs Control		DTLL vs Control		DTLL+Gem vs Control	
		Fold change	P value	Fold change	P value	Fold change	P value	Fold change	P value	Fold change	P value	Fold change	P value	Fold change	P value
ABCC1	P33527	0.878	5.89E-02	1.082	2.53E-01	0.942	4.90E-01	1.168	1.72E-01	1.206	6.22E-02	1.061	8.29E-02	1.026	4.88E-01
CDA	P32320	0.769	2.87E-02	0.867	7.19E-02	0.803	8.01E-02	0.639	8.45E-03	0.977	7.90E-01	1.043	5.02E-01	1.175	1.41E-01
CDC5L	Q99459	0.883	2.31E-02	1.076	1.03E-01	1.090	9.82E-02	1.082	8.58E-02	1.040	1.02E-01	0.863	8.75E-03	0.871	5.16E-03
CMPK1	P30085	1.229	1.67E-03	0.987	8.42E-01	0.877	5.30E-02	0.910	1.32E-01	1.012	7.14E-01	1.008	4.68E-01	1.039	1.76E-01
DCK	D6RFG8	0.899	1.93E-01	1.649	1.18E-03	0.996	9.51E-01	1.259	1.60E-02	1.234	5.18E-02	0.896	3.61E-01	0.877	6.48E-02
DCTD	P32321	1.062	3.05E-01	1.085	1.95E-01	1.062	3.36E-01	1.043	5.26E-01	1.026	1.27E-01	1.031	6.16E-02	1.068	6.99E-03
NT5C	Q8TCD5	1.232	4.31E-03	0.968	3.29E-01	1.023	5.30E-01	0.909	7.85E-02	1.006	8.67E-01	1.028	2.78E-01	1.087	7.16E-02
NT5C2	P49902	0.989	6.56E-01	1.107	1.92E-02	0.997	9.77E-01	1.190	1.54E-02	1.015	6.98E-01	1.067	6.97E-02	1.045	3.45E-02
NT5C3A	Q9H0P0	0.963	4.87E-01	1.646	2.79E-04	1.064	1.19E-01	1.122	2.84E-03	1.113	9.74E-02	0.971	5.76E-01	0.950	3.60E-01
RRM1	P23921	0.589	1.30E-03	1.897	4.37E-04	0.607	2.37E-03	0.713	6.15E-03	1.027	6.76E-01	0.578	2.71E-04	0.534	4.47E-05
RRM2	P31350	0.222	3.15E-06	6.409	5.57E-06	1.622	2.16E-04	4.028	1.92E-05	1.331	3.43E-03	0.473	1.15E-04	0.480	1.96E-05
SLC29A1	Q99808	0.712	5.77E-02	1.490	6.53E-02	0.569	3.71E-02	0.960	8.60E-01	1.221	3.50E-02	0.679	1.55E-02	0.574	3.09E-04
SMAD4	K7EIU8	0.017	1.69E-06	1.150	3.52E-01	7.260	4.40E-05	4.835	4.45E-05	1.013	9.61E-01	1.269	2.21E-01	1.315	7.04E-02

Note: Each cell sample in triplicates was collected and conducted in proteomic analysis. The average of area value was used for protein quantification.
The difference between groups was analyzed by T-test with two sides and adjusted using Benjamin Hochberg correction, and $p < 0.05$ was considered to be statistically significant.

Supplementary Table 4. Sequences of oligonucleotides for SMAD4-specific siRNAs and a scramble siRNA.

Name	DNA sequence
SMAD4 siRNA-1	5'-AGAUGAAUUGGAUUCUUUAdTdT-3'
SMAD4 siRNA-2	5'-GUGUGCAGUUGGAAUGUAAdTdT-3'
SMAD4 siRNA-3	5'-GUACUUCAUACCAUGCCGAdTdT-3'
SMAD4 siRNA-4	5'-CAUCCUAGUAAAUGUGUUAdTdT-3'
Scrambled siRNA (si-NC)	5'-ACGCGUAACGCGGGAAUUUdTdT-3'

Supplementary Table 5. Sequences of oligonucleotides for primers used for real time qRT-PCR.

Gene name	Forward primer	Reverse primer
SMAD4	5′ -TCCAGCCTCCCATTTCCAAT-3′	5′ -ACCTTGCTCTCTCAATGGCT-3′
TRIM33	5′ -AAATGCAAACCGAGGTCCCA-3′	5′ -CCAGGAGTGCATCATCGGAA-3′
P50	5′ -GCTTAGGAGGGAGAGCCCAC-3′	5′ -AGGACGTTGTGTTCCTTCCG-3′
P65	5′ -TTCTTTCGCCGAAGTCAGGG-3′	5′ -GCTGCTCGCTTGTCTTTTCG-3′
FADD	5′ -TCTACCTCCGAAGCGTCCTGAT-3′	5′ -AGGTGGTCTGTGGCTCACTCA-3′
BCL2	5′ -CTTTGAGTTCGGTGGGGTCA-3′	5′ -GAAATCAAACAGAGGCCGCA-3′
MCL1	5′ -AACGCGGTAATCGGACTCAA-3′	5′ -CCTCCTTCTCCGTAGCCAAA-3′
BAX	5′ -TCATGGGCTGGACATTGGAC-3′	5′ -GCGTCCCAAAGTAGGAGAGG-3′
GAPDH	5′ -GCAAATTCCATGGCACCGT-3′	5′ - TCGCCCCACTTGATTTTGG-3′
