# Supplementary material for: An EGFR/HER2-targeted conjugate sensitizes gemcitabine-sensitive and resistant pancreatic cancer through different SMAD4-mediated mechanisms
Source: Nat Commun. 2022 Sep 20;13:5506. doi: 10.1038/s41467-022-33037-x (PMC9489697; doi:10.1038/s41467-022-33037-x)
Supplement: Supplementary file 3 — Reporting Summary [file 41467_2022_33037_MOESM3_ESM.pdf]

nature portfolio

Corresponding author(s): Liang Li & Rongguang Shao

Last updated by author(s): May 15, 2022

Reporting Summary

Nature Portfolio wishes to improve the reproducibility of the work that we publish. This form provides structure for consistency and transparency in reporting. For further information on Nature Portfolio policies, see our Editorial Policies and the Editorial Policy Checklist.

Statistics

For all statistical analyses, confirm that the following items are present in the figure legend, table legend, main text, or Methods section.

n/a	Confirmed	
☐	☒	The exact sample size (n) for each experimental group/condition, given as a discrete number and unit of measurement
☐	☒	A statement on whether measurements were taken from distinct samples or whether the same sample was measured repeatedly
☐	☒	The statistical test(s) used AND whether they are one- or two-sided<br>*Only common tests should be described solely by name; describe more complex techniques in the Methods section.*
☐	☒	A description of all covariates tested
☐	☒	A description of any assumptions or corrections, such as tests of normality and adjustment for multiple comparisons
☐	☒	A full description of the statistical parameters including central tendency (e.g. means) or other basic estimates (e.g. regression coefficient) AND variation (e.g. standard deviation) or associated estimates of uncertainty (e.g. confidence intervals)
☐	☒	For null hypothesis testing, the test statistic (e.g. F, t, r) with confidence intervals, effect sizes, degrees of freedom and P value noted<br>*Give P values as exact values whenever suitable.*
☐	☒	For Bayesian analysis, information on the choice of priors and Markov chain Monte Carlo settings
☐	☒	For hierarchical and complex designs, identification of the appropriate level for tests and full reporting of outcomes
☐	☒	Estimates of effect sizes (e.g. Cohen's d, Pearson's r), indicating how they were calculated

Our web collection on statistics for biologists contains articles on many of the points above.

Software and code

Policy information about availability of computer code

Data collection	No software was used to collect data.
Data analysis	Band intensities were quantified using ImageJ (version 1.51j8) . The combination index (CI) of both drugs was calculated using the Chou–Talalay method and the CompuSyn software (Version 1.0) . Cell cycle data was analyzed by Becton Dickinson cell fit software (version 2.0) and FlowJo (version 10) software. Sequence of wild-type Smad4 and mutant Smad4 R100T constructs were analyzed with SnapGene (version 6.0.2). Statistical analysis was performed using SPSS (version 13.0) software.

For manuscripts utilizing custom algorithms or software that are central to the research but not yet described in published literature, software must be made available to editors and reviewers. We strongly encourage code deposition in a community repository (e.g. GitHub). See the Nature Portfolio guidelines for submitting code & software for further information.

Data

Policy information about availability of data

All manuscripts must include a data availability statement. This statement should provide the following information, where applicable:

- Accession codes, unique identifiers, or web links for publicly available datasets
- A description of any restrictions on data availability
- For clinical datasets or third party data, please ensure that the statement adheres to our policy

The producer clones of DTLP (the precursor of DTLL) fusion protein in this study have been deposited into the China Pharmaceutical Culture Collection (CPCC) under Accession Number CCPC101501 [http://www.cpcc.ac.cn/]. All the proteomic raw data and the results files in this study have been deposited in the iProX Consortium database under accession code PXD031977 [https://www.iprox.org/]. The raw data used in this study were downloaded from the TCGA-PAAD Project of TCGA public

datasets on the website (https://portal.gdc.cancer.gov/repository), including clinical information on gemcitabine treatment and SMAD4 expression data of pancreatic cancer patients, which are provided in Supplementary data 1 (TCGA-PAAD+clinical_drug.txt) and Supplementary data 2 (TCGA-PAAD.htseq_fpkm.tsv).

Field-specific reporting

Please select the one below that is the best fit for your research. If you are not sure, read the appropriate sections before making your selection.

☒ Life sciences ☐ Behavioural & social sciences ☐ Ecological, evolutionary & environmental sciences

For a reference copy of the document with all sections, see nature.com/documents/nr-reporting-summary-flat.pdf

Life sciences study design

All studies must disclose on these points even when the disclosure is negative.

Sample size	Sample sizes were chosen based on the experimental variability and amount of samples required for statistical analysis. For in vitro experiments, at least three biological replicates were achieved for all the experiments. The number of animals allocated for in vivo studies were 3-6 in each experimental group, which is sufficient for a minimal data set underlying the figures provided in the paper in order to support and interpret our central findings. No statistical method was used to predetermine sample size in animal studies. Sample sizes are indicated in the figure legends and main text.
Data exclusions	No data were exluded from the analyses.
Replication	The results from in vitro tests were obtained from three independent experiments, and the data are presented as the mean ± SD (n =3). To evaluate in vivo efficacy by cell line-derived xenograft (CDX) models of human pancreatic cancer, the data are presented as the mean ± SEM (n =6 for AsPC-1, n = 5 for MIA PaCa-2 tumors and BxPC-3 tumors). For in vivo evaluation by PDX models, the data are presented as the mean ± SEM (n =3 for PA1233 and n = 4 for PA3142 tumors). All attempts at replication were successful.
Randomization	All animals during in vivo evaluation by CDX and PDX models were randomly allocated into experimental groups. The mice were earmarked before grouping and then were randomly separated into groups by an independent person.
Blinding	Animal studies were blindly investigated for allocation of experimental groups, administration, collection and /or analysis. The investigators were blinded to group allocation during data collection and/or analysis.

Reporting for specific materials, systems and methods

We require information from authors about some types of materials, experimental systems and methods used in many studies. Here, indicate whether each material, system or method listed is relevant to your study. If you are not sure if a list item applies to your research, read the appropriate section before selecting a response.

Materials & experimental systems

n/a	Involved in the study	
☐	☒	Antibodies
☐	☒	Eukaryotic cell lines
☒	☐	Palaeontology and archaeology
☐	☒	Animals and other organisms
☒	☐	Human research participants
☒	☐	Clinical data
☒	☐	Dual use research of concern

Methods

n/a	Involved in the study	
☒	☐	ChIP-seq
☐	☒	Flow cytometry
☒	☐	MRI-based neuroimaging

Antibodies

Antibodies used	Primary antibodies: Phospho-EGF Receptor (Tyr1068) (D7A5) Rabbit mAb (#3777), EGF Receptor (1F4) Mouse mAb (#2239), Phospho-HER2/ErbB2 (Tyr1221/1222) (6B12) Rabbit mAb (#2243), HER2/ErbB2 (29D8) Rabbit mAb (#2165), Phospho-Akt (Ser473) (D9E) Rabbit mAb (#4060), Akt (pan) (C67E7) Rabbit mAb (#4691), Phospho-mTOR (Ser2448) (D9C2) Rabbit mAb (#5536), mTOR (7C10) Rabbit mAb (#2983), Phospho-p44/42 MAPK (Erk1/2) (Thr202/Tyr204) (D13.14.4E) Rabbit mAb (#4370), p44/42 MAPK (Erk1/2) (137F5) Rabbit mAb (#4695), PD-L1 (E1L3N®) XP® Rabbit mAb (#13684), Phospho-Bcl-2 (Ser70) (5H2) Rabbit mAb (#2827), Phospho-Histone rH2AX (Ser139) Antibody (#2577), Cyclin D1 (E3P5S) Rabbit mAb (#55506), TGF-β Antibody (#3711), Phospho-SMAD2 (Ser465/467) (138D4) Rabbit mAb (#3108), SMAD2 (D43B4) Rabbit mAb (#5339), SMAD2/3 (D7G7) Rabbit mAb (#8685), Phospho-SMAD3 (Ser423/425) (C25A9) Rabbit mAb (#9520), SMAD3 (C67H9) Rabbit mAb (#9523), SMAD4 (D3R4N) Rabbit mAb (#46535), TRIM33 (D7U4F) Rabbit mAb (#90051), Cyclin D3 (DCS22) Mouse mAb (#2936), Cyclin B1 (D5C10) XP® Rabbit mAb (#12231), Cyclin E2 Antibody (#4132), CDK2 (78B2) Rabbit mAb (#2546), CDK4 (D9G3E) Rabbit mAb (#12790), NF-κB1 p105/p50 (D7H5M) Rabbit mAb (#12540), Phospho-NF-κB p65 (Ser536) (93H1) Rabbit mAb (#3033), NF-κB p65 (D14E12) XP® Rabbit mAb (#8242), Phospho-cdc2 (Tyr15) (10A11) Rabbit mAb (#4539), Phospho-Wee1 (Ser642) (D47G5) Rabbit mAb (#4910), p21 Waf1/Cip1 (12D1) Rabbit mAb (#2947), p27 Kip1 (D69C12) XP® Rabbit mAb (#3686), Cleaved Caspase-8 (Asp374) (18C8) Rabbit mAb (#9496), Bcl-2 (124) Mouse mAb (#15071), MCL1 (D2W9E)

Rabbit mAb (#94296), Bax (D2E11) Rabbit mAb (#5023), β-actin (8H10D10) Mouse mAb (#3700) were purchased from Cell Signaling (Beverly, MA, USA) for Western blot (1:1,000 dilution). SMAD7 Mouse Monoclonal antibody (66478-1-Ig, 1:1500 dilution), Lamin B1 Mouse Monoclonal antibody (66095-1-Ig, 1:1000 dilution) were purchased from Proteintech (Thermo Fisher Scientific, Waltham, MA, USA) for Western blot. Anti-FADD Antibody (G-4) (sc-271748, 1:500 dilution) was purchased from Santa Cruz Biotechnology for Western blot. Rabbit anti-Ki67 antibody (ab92742, 1:500 dilution) was purchased from Abcam for IHC.
Secondary antibodies:
Horseradish peroxidase (HRP)-conjugated anti-rabbit IgG and horseradish peroxidase (HRP)-conjugated anti-mouse IgG were purchased from Zhongshanjinqiao (Beijing, China) for Western blot (1:5,000 dilution).
Goat anti-Rabbit IgG (H+L) Highly Cross-Adsorbed Secondary Antibody, Alexa Fluor™ 488 (A-11034, 1:500 dilution) was purchased from Invitrogen for Immunofluorescence.

Validation

In this study, only commercially available antibodies were used. Each primary antibody was validated for the species and aplication on the manufacturer's website.

Eukaryotic cell lines

Policy information about cell lines

Cell line source(s)

Human pancreatic carcinoma cell lines AsPC-1, MIA PaCa-2, BxPC-3, PANC-1, CFPAC-1, Panc0403, HuPT-3 and SU86.86 used were purchased from ATCC, and obtained from Dr Liewei Wang from the Department of Molecular Pharmacology of Experimental Therapeutics, Mayo clinic.

Authentication

None of the cell lines used were authenticated as those were obtained from Dr. Liewei Wang who had purchased those cell lines from ATCC.

Mycoplasma contamination

The cell lilnes were not tested for mycoplasm contamination.

Commonly misidentified lines
(See ICLAC register)

No commonly misidentified cell lines were used in this study.

Animals and other organisms

Policy information about studies involving animals; ARRIVE guidelines recommended for reporting animal research

Laboratory animals

Five weeks old female BALB/c nude mice used in the study were inoculated with human pancreatic cancer cells (AsPC-1 cells, MIA PaCa-2 cells, BxPC-3 cells) or human pancreatic tumor tissue samples (patient-derived xenograft (PDX) models of PA1233 and PA3142)subcutaneously. All experiments were conducted under specific pathogen-free (SPF) conditions. Mice were housed in groups of 5 mice per individually ventilated cage in a 12h light/dark cycle, with a temperature of 20±2°C) and relative humidity of 55 % ± 15. All mice had access to food and water ad libitum.

Wild animals

No wild animals were used in the study.

Field-collected samples

No field collected samples were used in the study.

Ethics oversight

Animal protocols were approved by the Institutional Animal Care and Use Committee of the Institute of Medicinal Biotechnology (IMB), Chinese Academy of Medical Sciences & Peking Union Medical College (CAMS & PUMC).

Note that full information on the approval of the study protocol must also be provided in the manuscript.

Flow Cytometry

Plots

Confirm that:

☒ The axis labels state the marker and fluorochrome used (e.g. CD4-FITC).

☒ The axis scales are clearly visible. Include numbers along axes only for bottom left plot of group (a 'group' is an analysis of identical markers).

☒ All plots are contour plots with outliers or pseudocolor plots.

☒ A numerical value for number of cells or percentage (with statistics) is provided.

Methodology

Sample preparation

Pancreatic carcinoma AsPC-1, MIA PaCa-2 and BxPC-3 cells were treated and fixed with pre-cold 70% ethanol, Then the cells were incubated with 100μg/ml RNase A (Beyotime Technologies, Shanghai, China) and 50μg/ml PI at 37 °C for 30 min, and the cell-cycle distribution was analyzed on a FACScan flow cytometer.

Instrument

ACS Calibur flow cytometry used in the study was made by BD Biosciences, San Diego, CA, USA.

Software

Becton Dickinson cell fit software (version 2.0) and FlowJo (version 10) software

Cell population abundance

In the flow cytometry-based cell cycle experiments, >10000 cells were collected per population. The purity of each

Cell population abundance	population was determined by using a minimum of 5 colors, which allows precise population identification by direct or back-gating approaches.
Gating strategy	We excluded debris by forward scatter (FSC)/side scatter (SSC) plot and doublet drops by FSC-hight /FSC-width and SSC-hight/ SSC-width plots.

☐ Tick this box to confirm that a figure exemplifying the gating strategy is provided in the Supplementary Information.
