# Supplementary material for: An EGFR/HER2-targeted conjugate sensitizes gemcitabine-sensitive and resistant pancreatic cancer through different SMAD4-mediated mechanisms
Source: Nat Commun. 2022 Sep 20;13:5506. doi: 10.1038/s41467-022-33037-x (PMC9489697; doi:10.1038/s41467-022-33037-x)
Supplement: Supplementary file 4 — Description of Additional Supplementary Files [file 41467_2022_33037_MOESM4_ESM.pdf]

Description of Additional Supplementary Files

File Name: Supplementary Data 1
Description: The raw data used for clinical information on gemcitabine treatment in this study were downloaded from the TCGA-PAAD Project of TCGA public datasets

File Name: Supplementary Data 2
Description: The raw data used for SMAD4 expression data of pancreatic cancer patients in this study were downloaded from the TCGA-PAAD Project of TCGA public datasets

File Name: Supplementary Data 3
Description: All the proteomic raw data of BxPC3-Mut and -WT cells with of without drug treatments in this study
